# Supplementary figures and images for: Supervised machine learning reveals introgressed loci in the genomes of Drosophila simulans and D. sechellia
Source: PLoS Genet. 2018 Apr 23;14(4):e1007341. doi: 10.1371/journal.pgen.1007341 (PMC5933812; doi:10.1371/journal.pgen.1007341)

Figure S1

No introgression

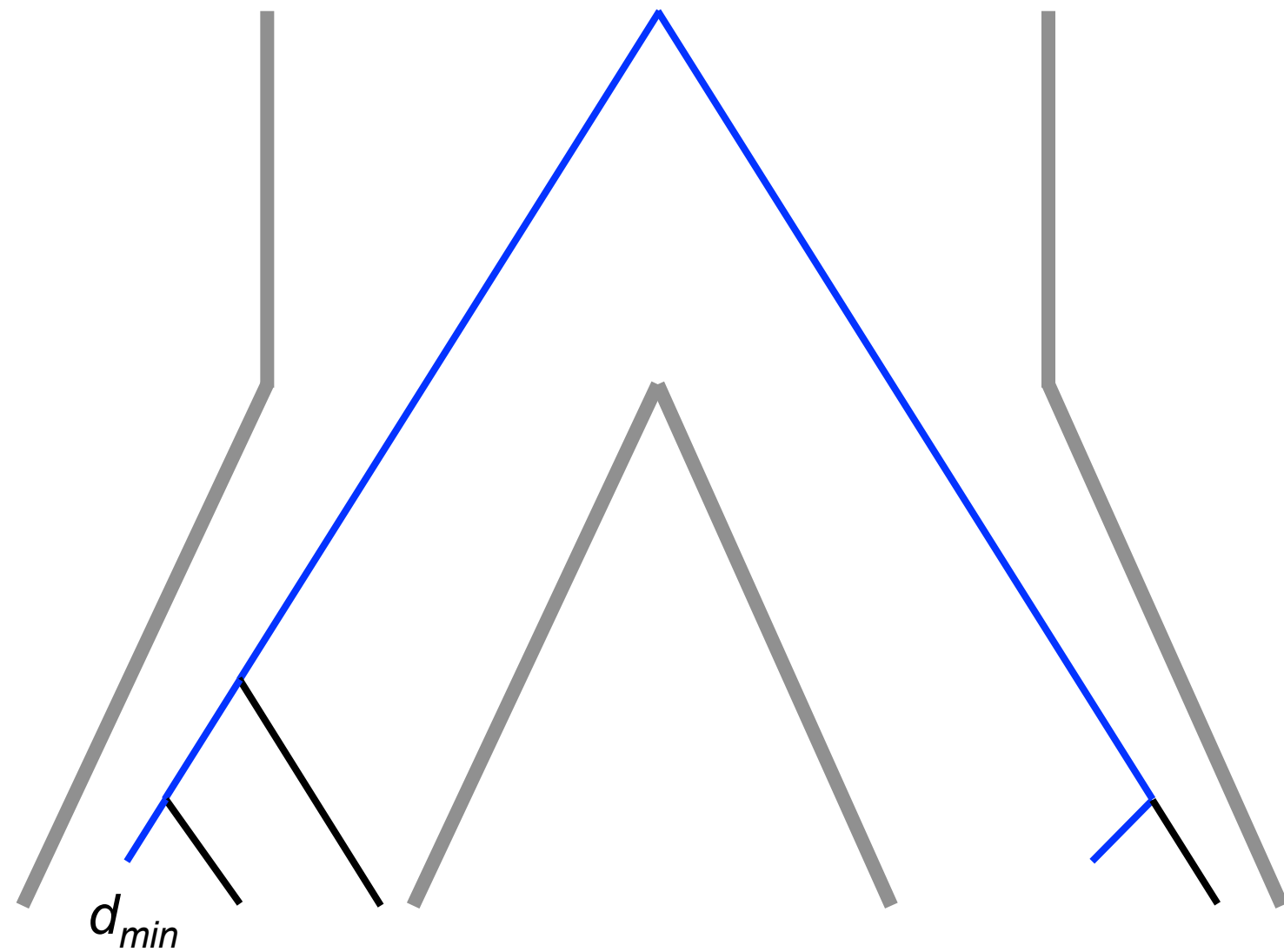

Introgression

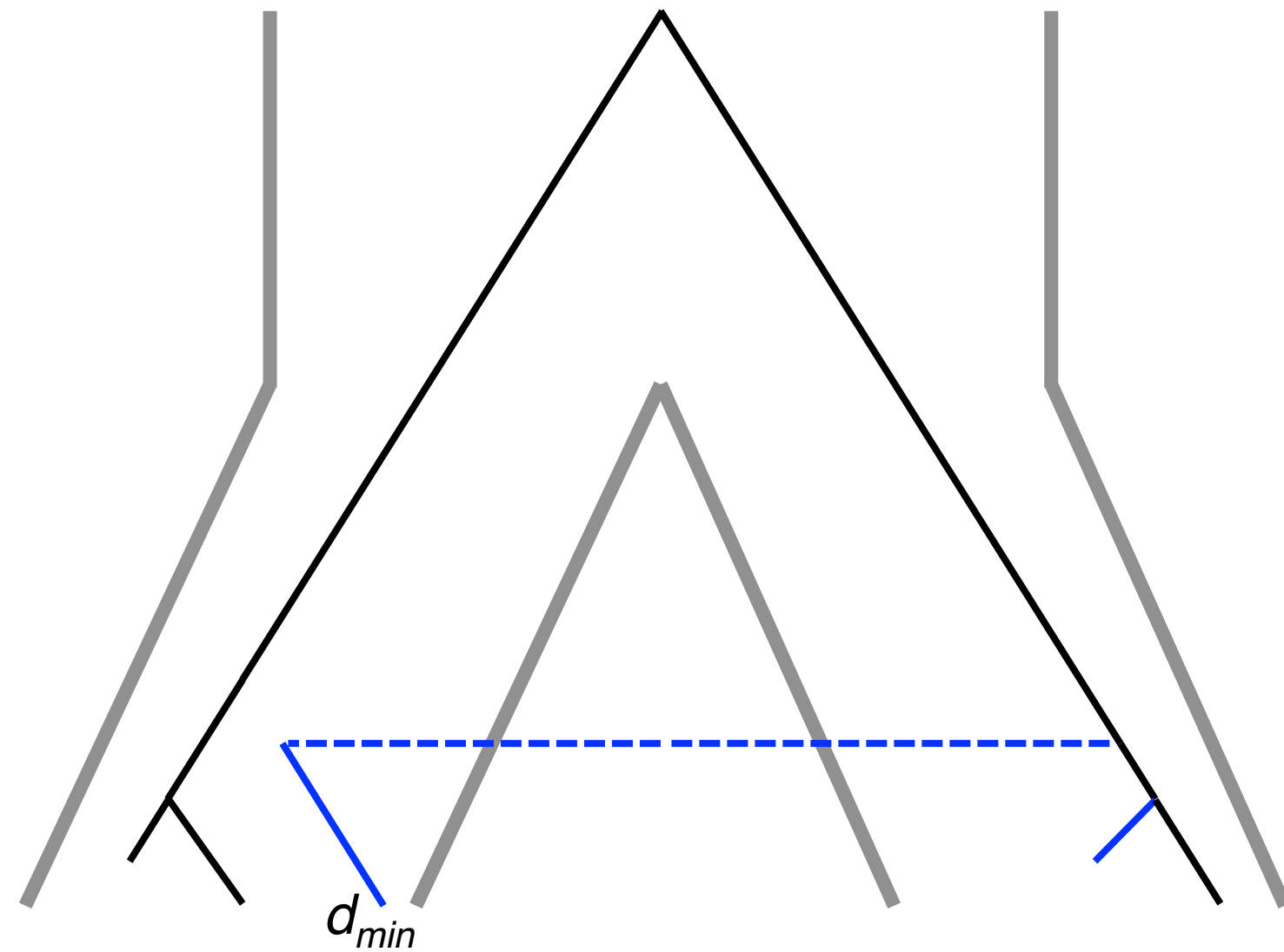

Supplement: S1 Fig — (PDF) [file pgen.1007341.s001.pdf]

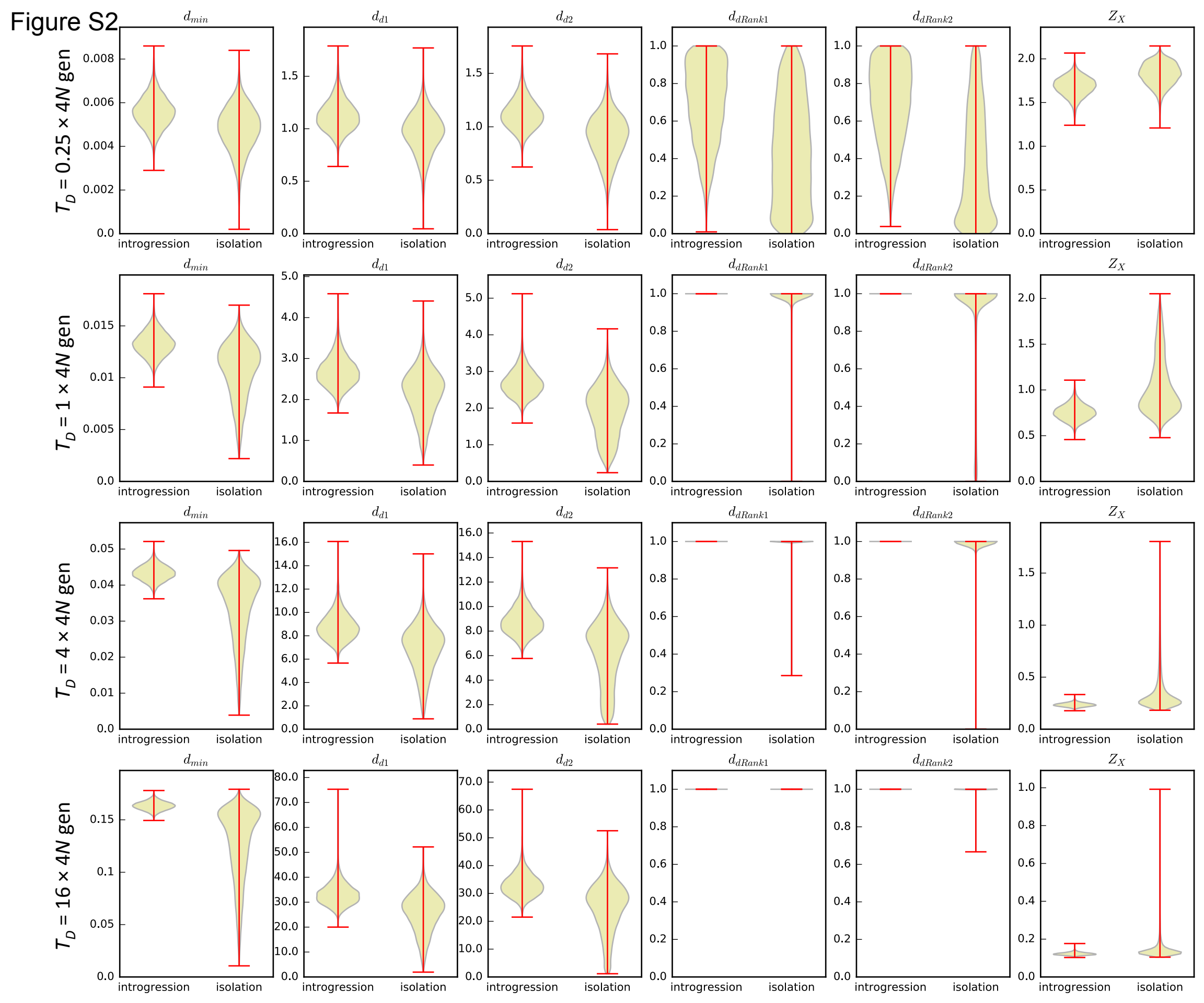

Supplement: S2 Fig — The values of these statistics were obtained from the training data sets described in the Materials and Methods. (PDF) [file pgen.1007341.s002.pdf]

Figure S3

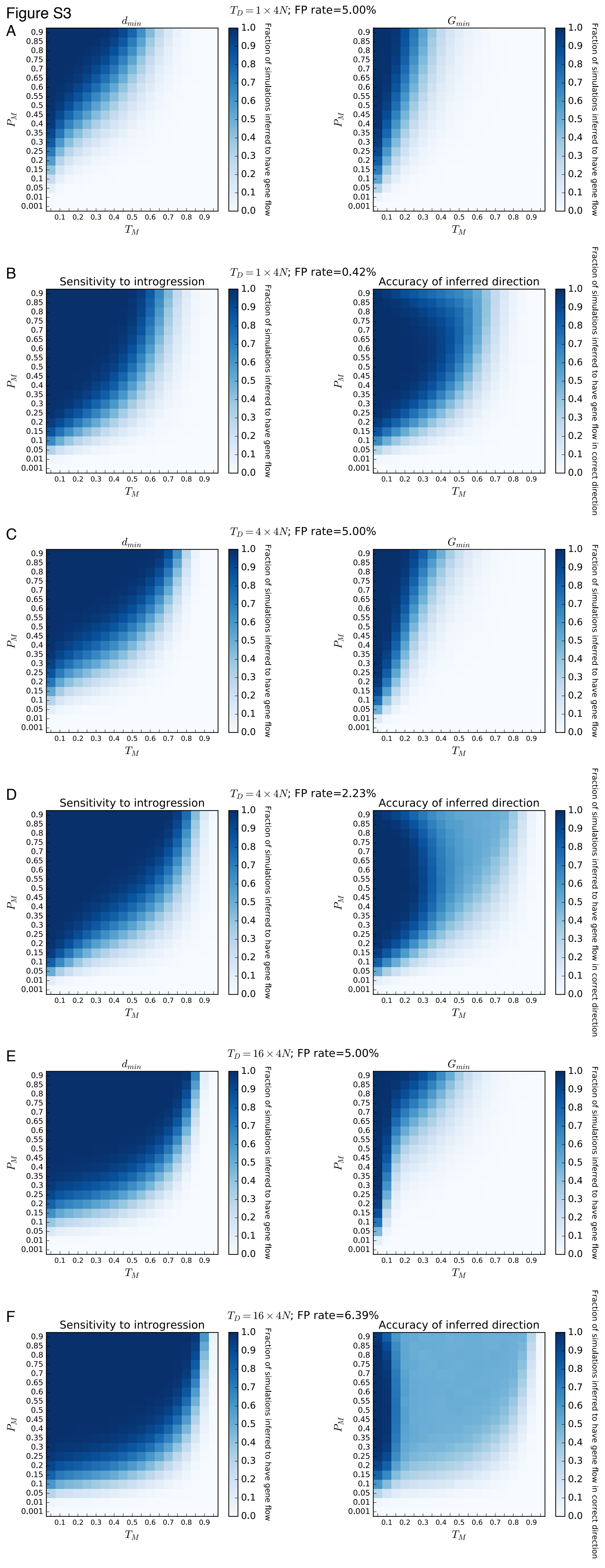

Supplement: S3 Fig — Same as Fig 1, but for other values of TD. (A) Accuracy for dmin and Gmin when TD = 1×4N generations. (B) Accuracy of FILET when TD = 1×4N. (C) and (D) show the same when TD = 4×4N. (E) and (F) show the same when 16×4N. (PDF) [file pgen.1007341.s003.pdf]

Figure S4

A

$$T_D = 0.25 \times 4N$$

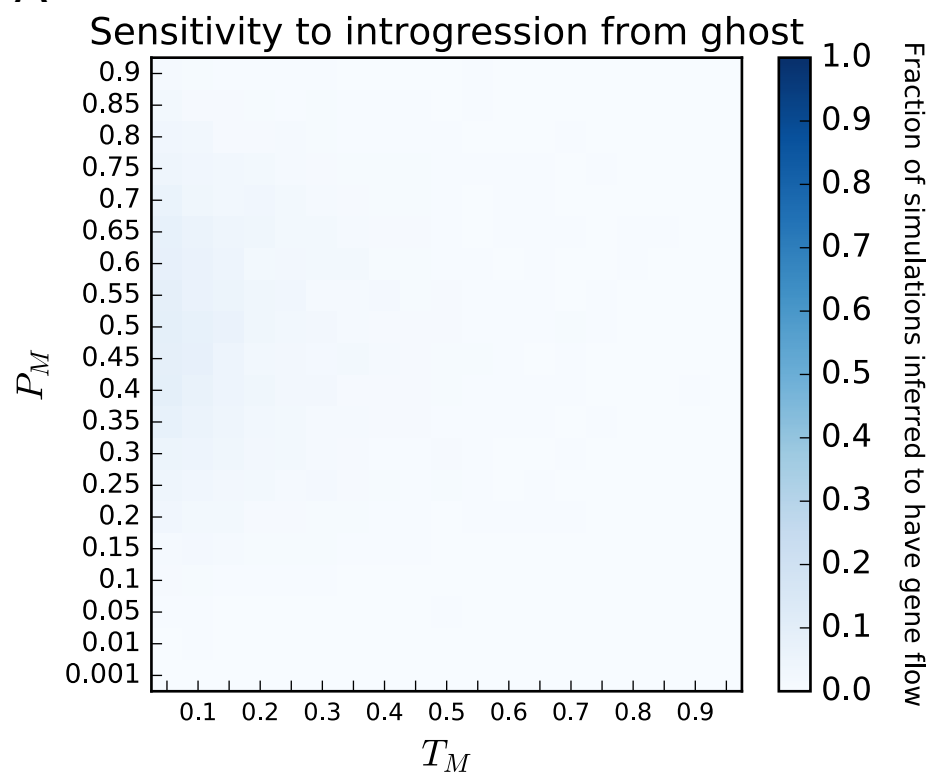

B

$$T_D = 1 \times 4N$$

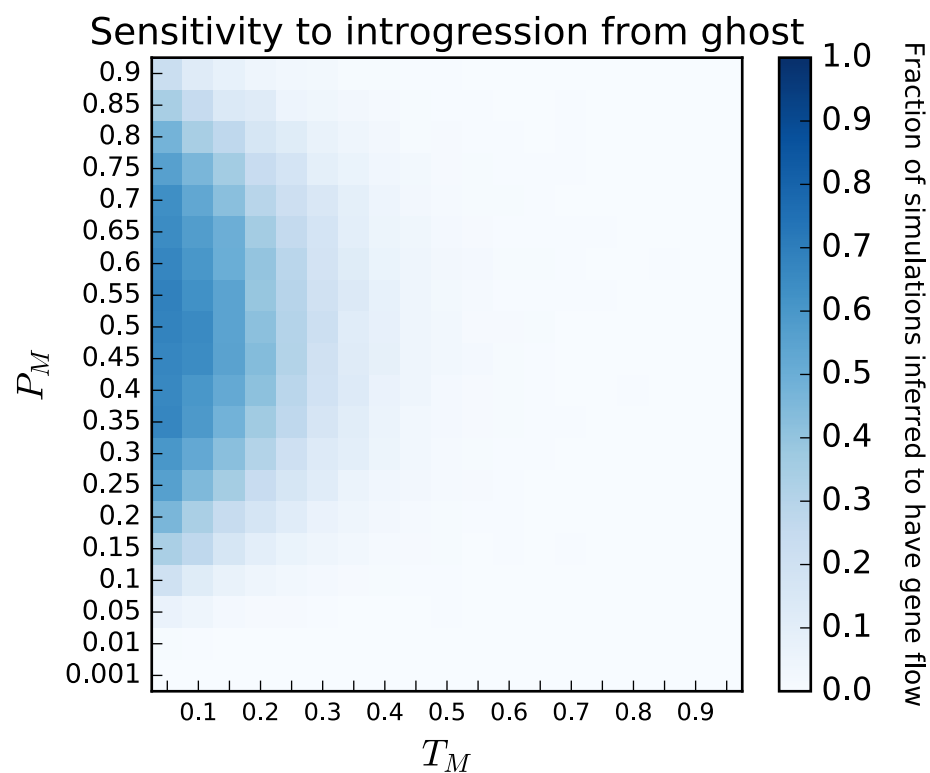

C

$$T_D = 4 \times 4N$$

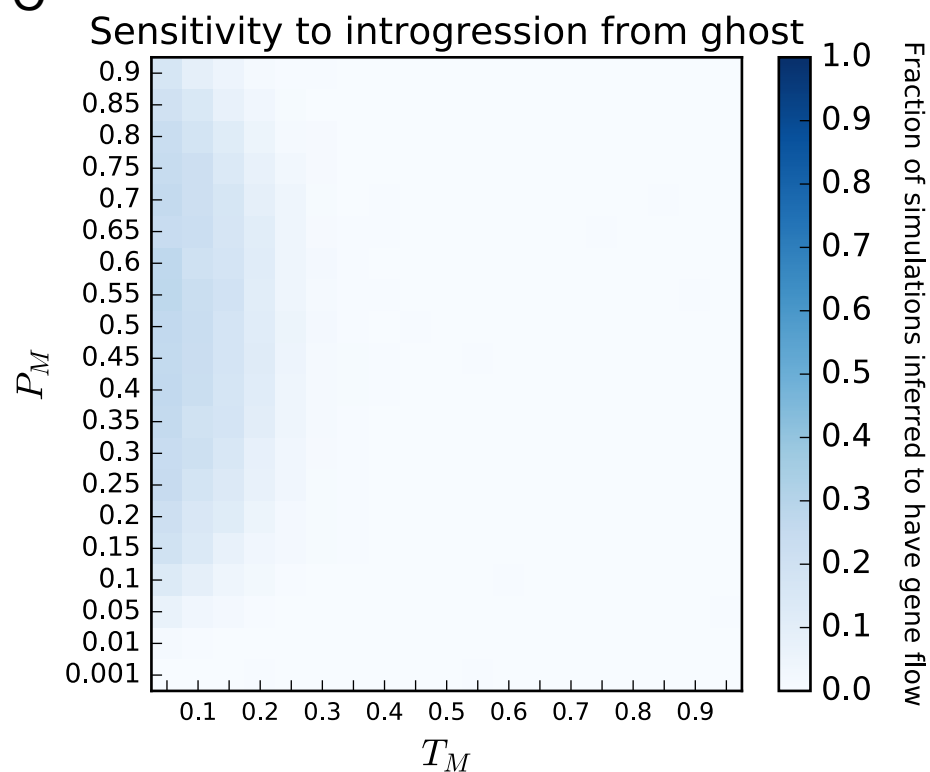

D

$$T_D = 16 \times 4N$$

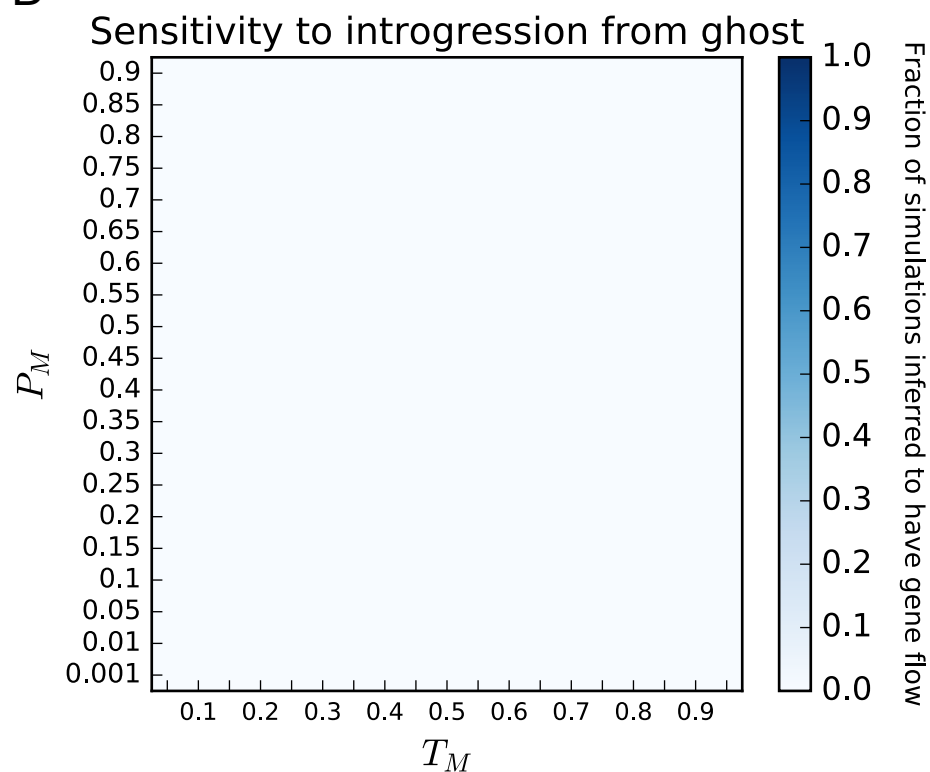

Supplement: S4 Fig — (A) Sensitivity when TD = 0.25×4N generations. (B) Sensitivity when TD = 1×4N generations. (C) TD = 4×4N generations. (D) TD = 16×4N. (PDF) [file pgen.1007341.s004.pdf]

Figure S5

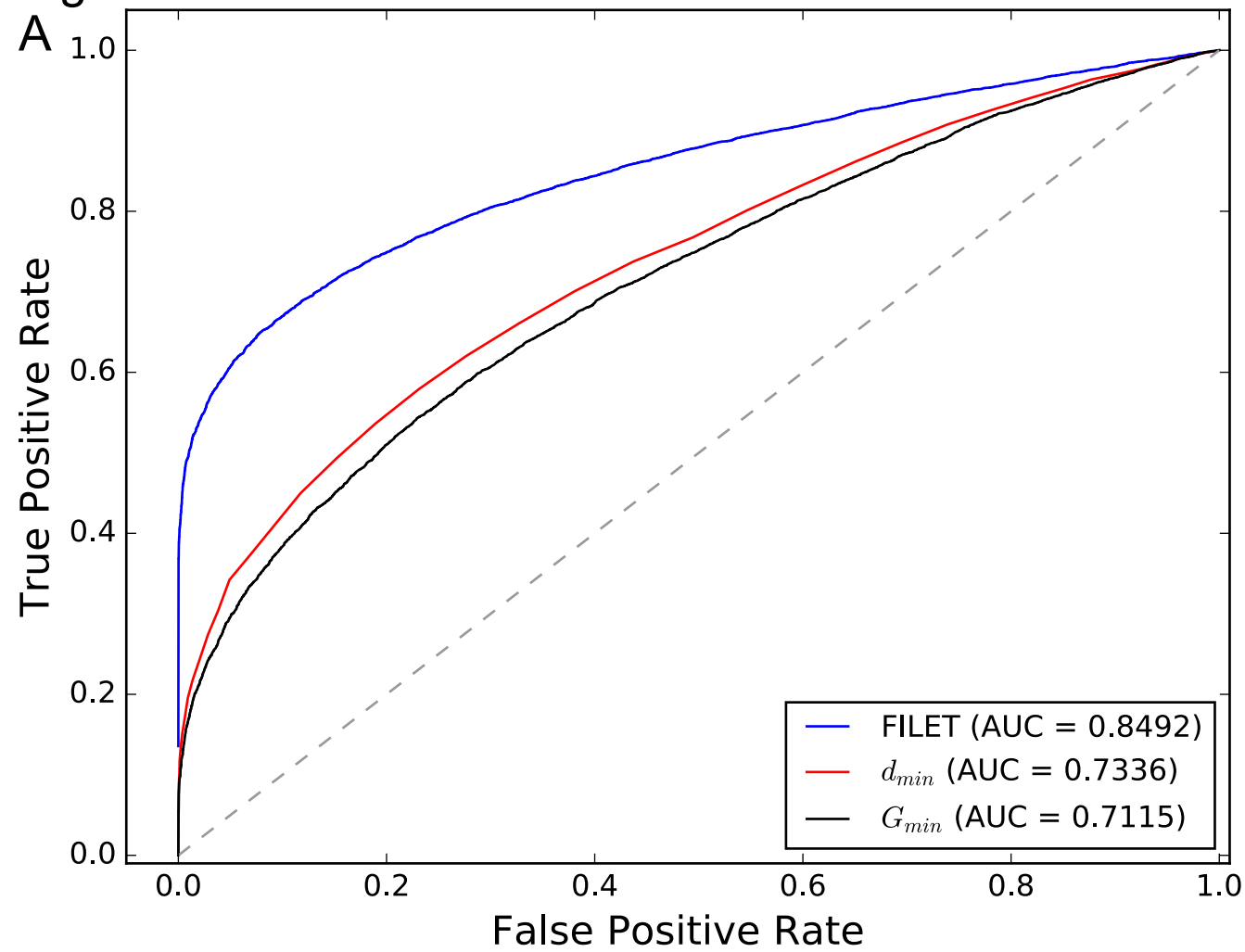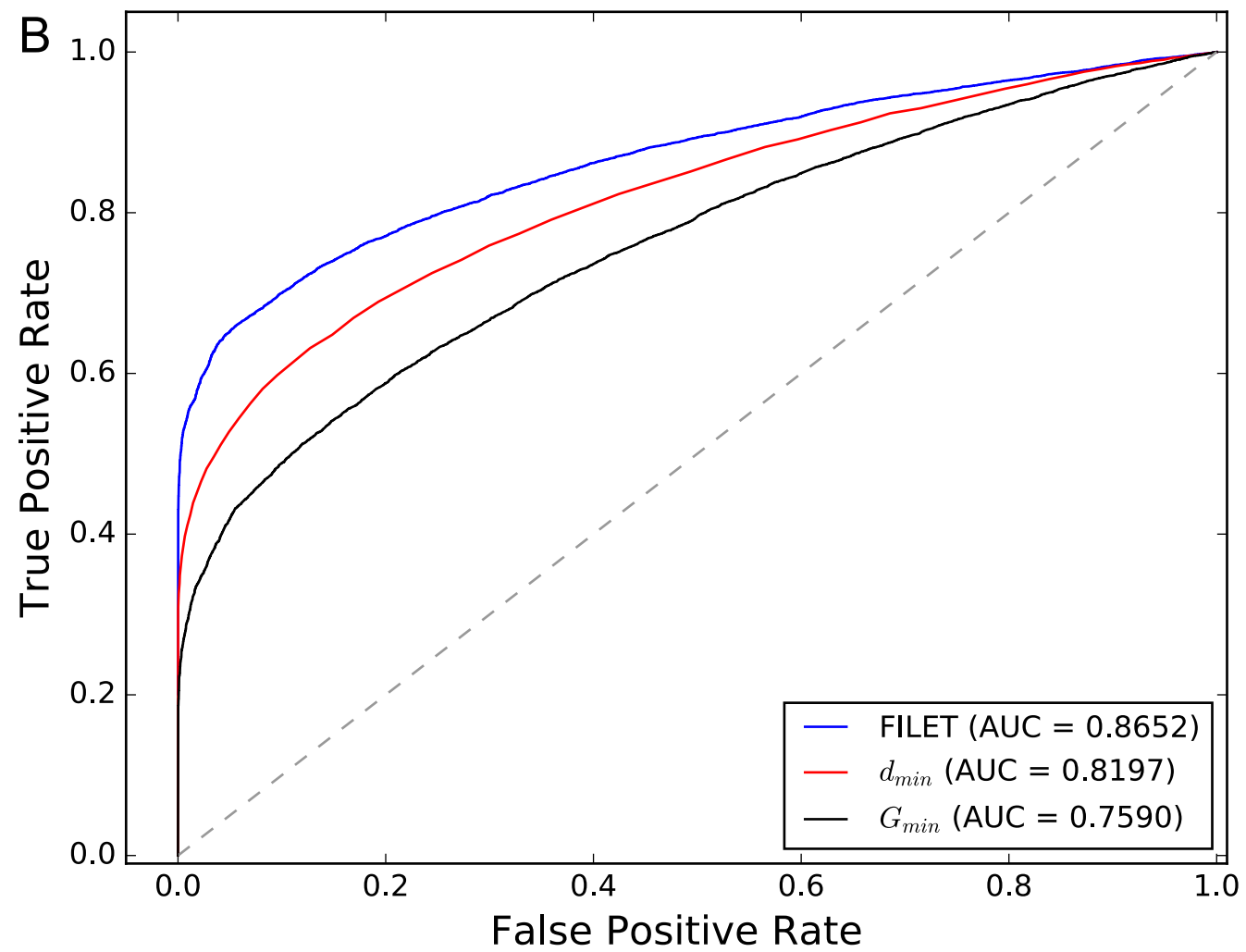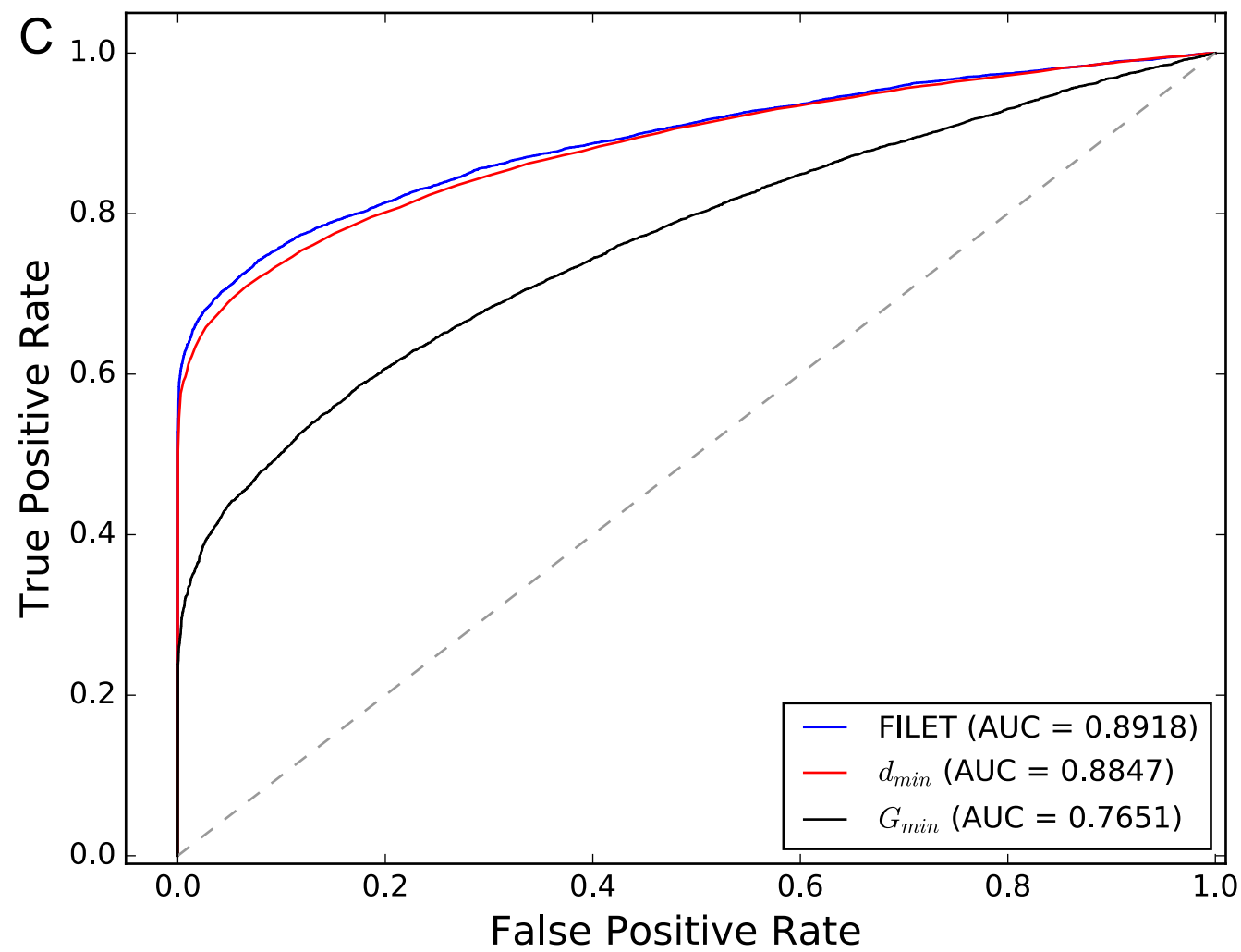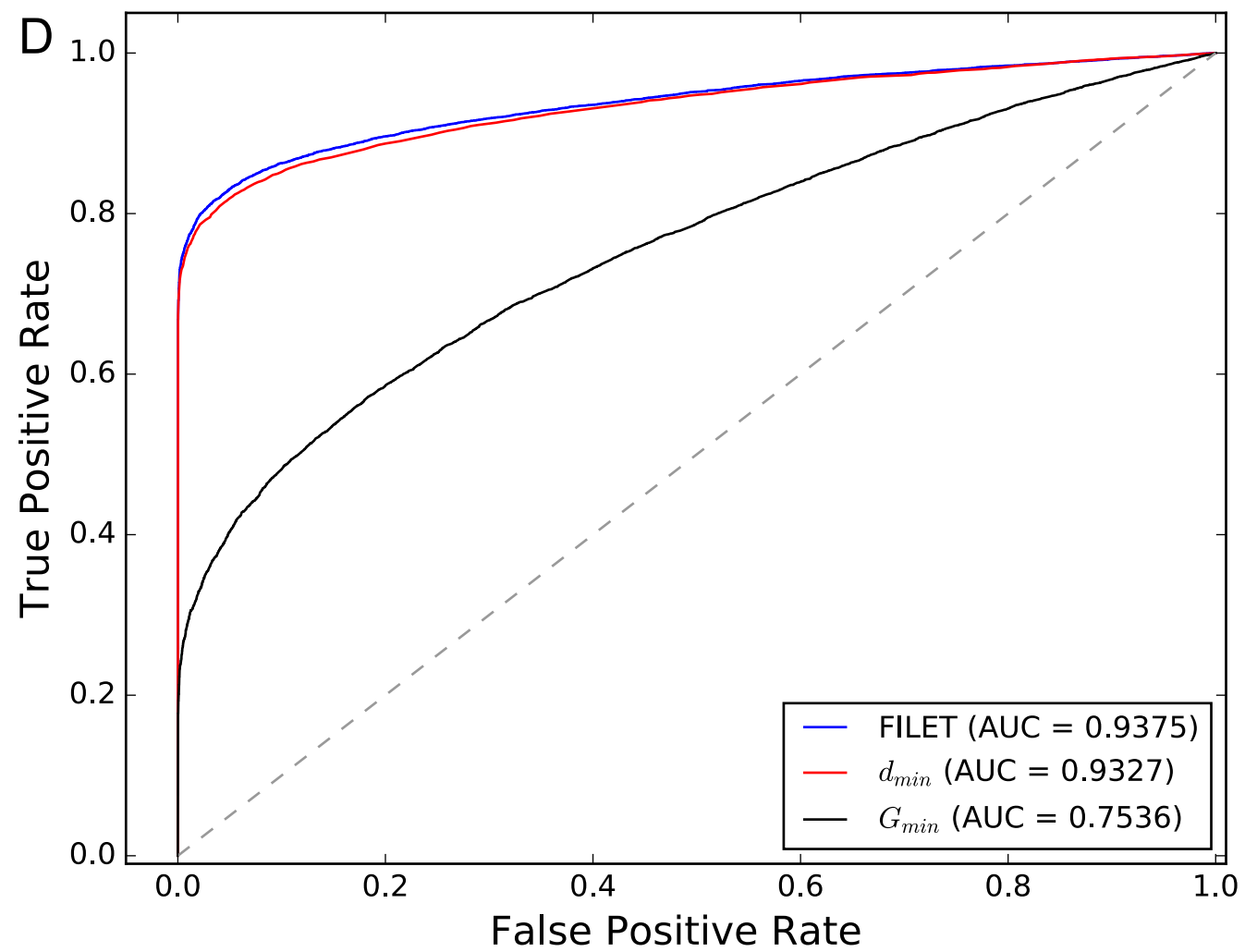

Supplement: S5 Fig — In order to generate these curves we transformed the classification task into a binary one: discriminating between isolation and introgression in either direction. (A) TD = 0.25×4N generations. (B) TD = 1×4N generations. (C) TD = 4×4N. (D) TD = 16×4N. Training and test sets for these problems contained equal numbers of examples of introgression from population 1 into 2 and introgression from population 2 into 1. (PDF) [file pgen.1007341.s005.pdf]

Figure S6

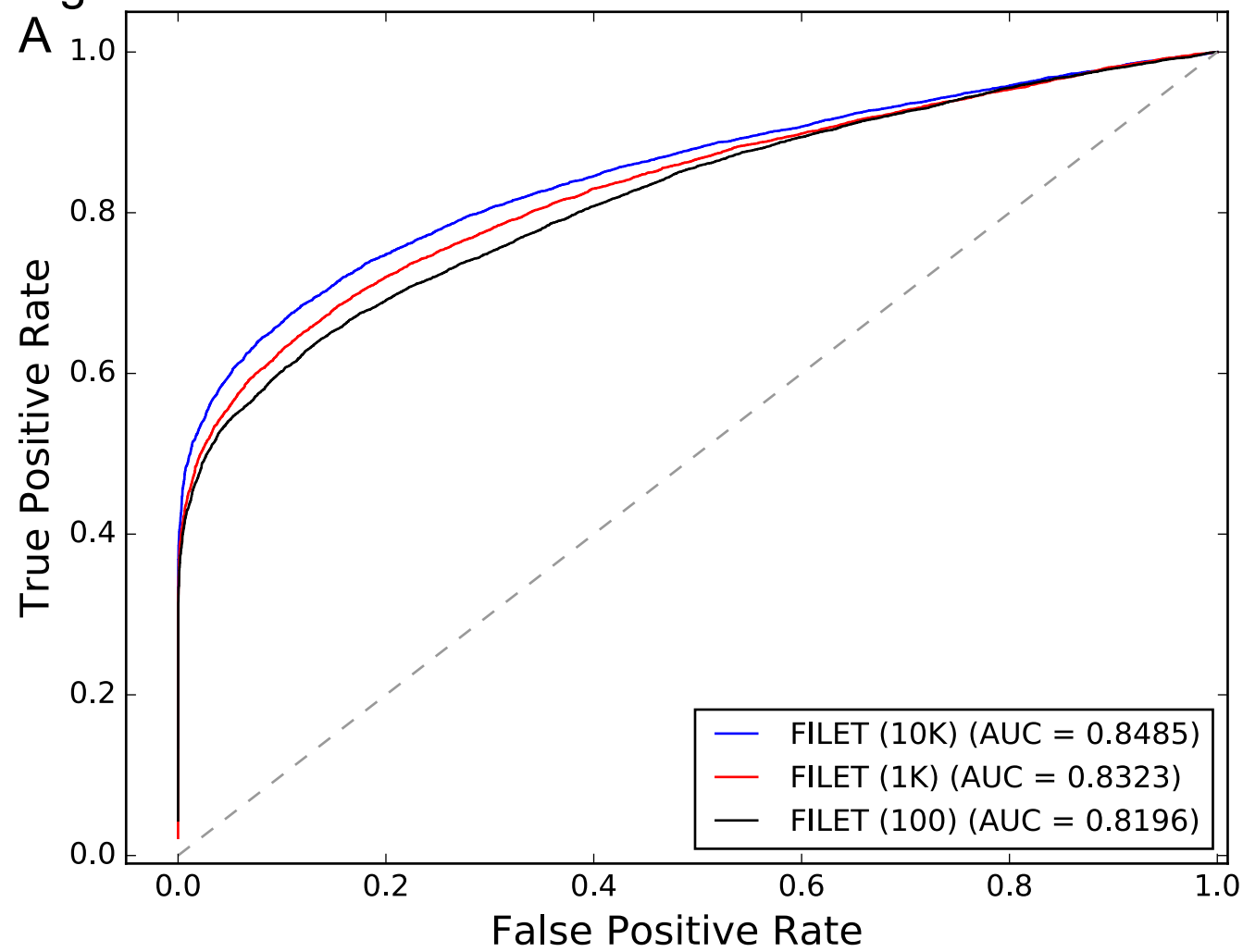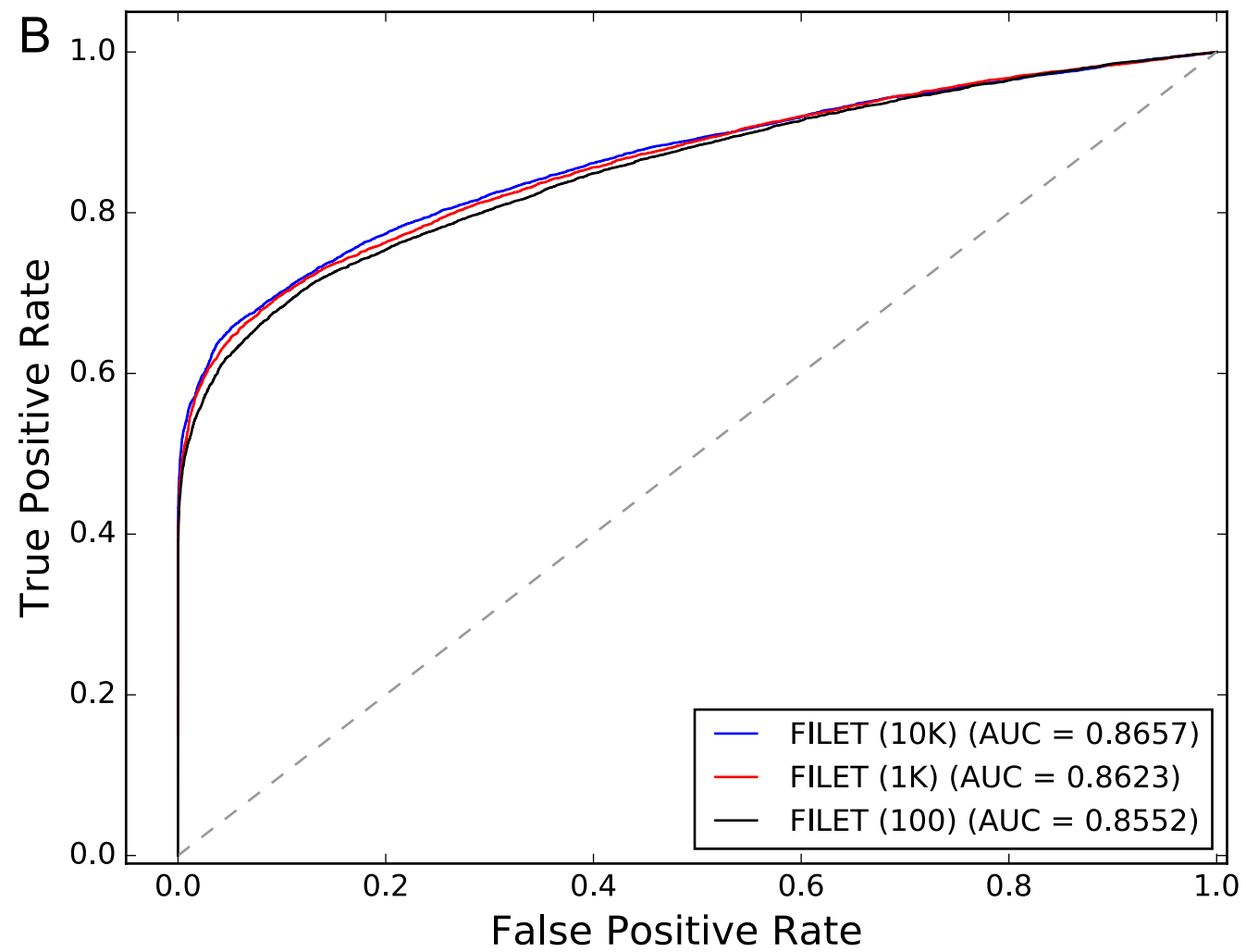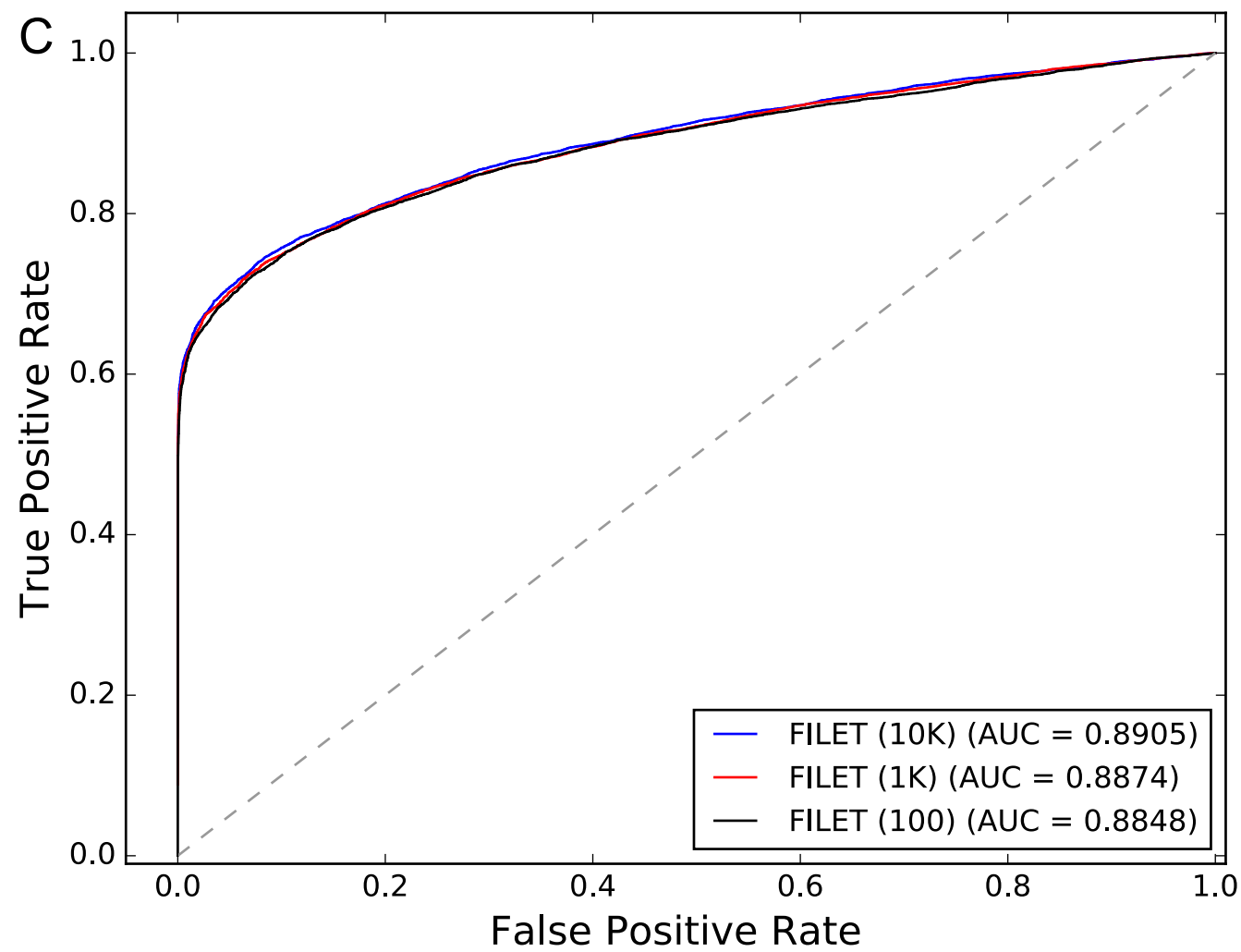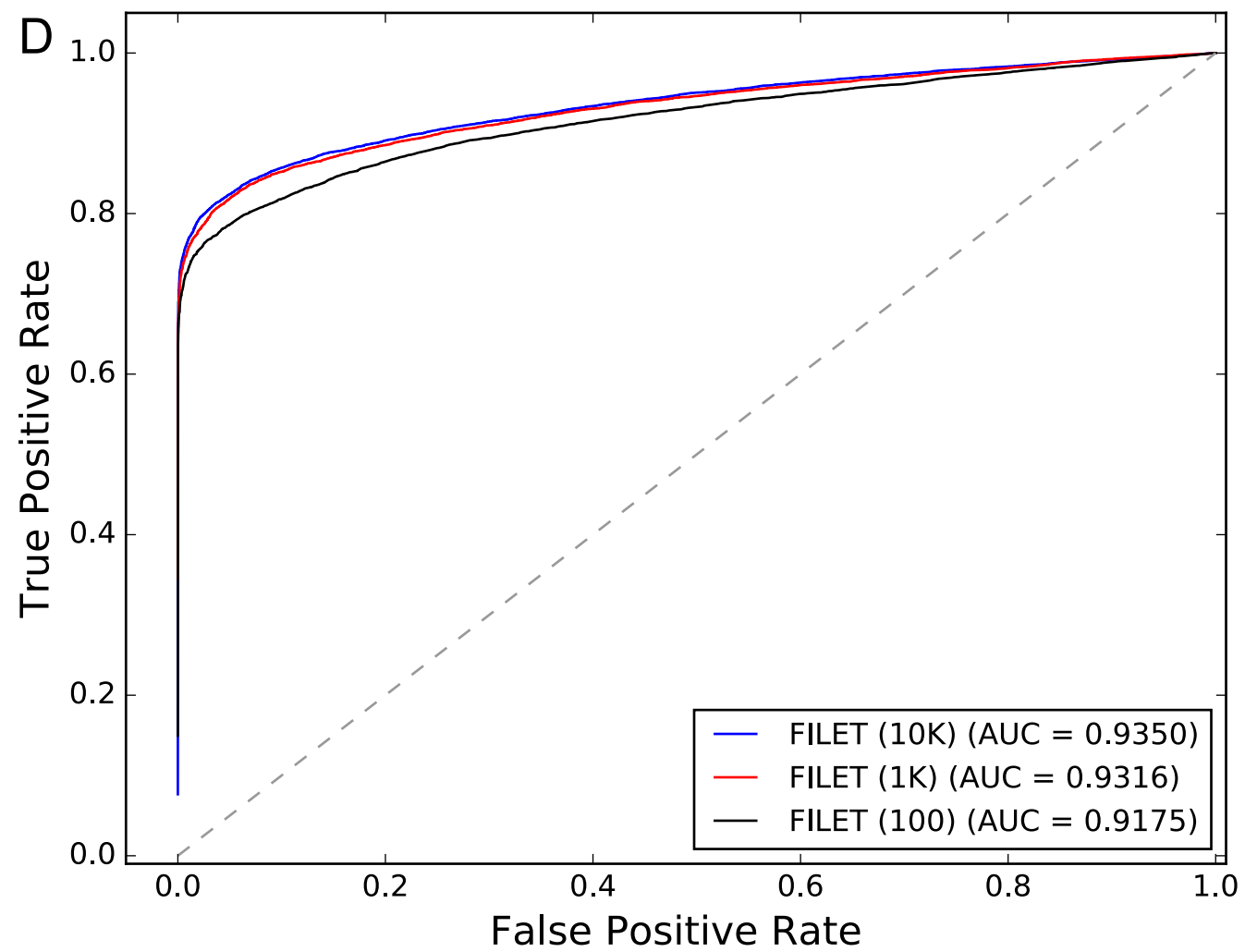

Supplement: S6 Fig — (A) TD = 0.25×4N generations. (B) TD = 1×4N generations. (C) TD = 4×4N. (D) TD = 16×4N. (PDF) [file pgen.1007341.s006.pdf]

Figure S7

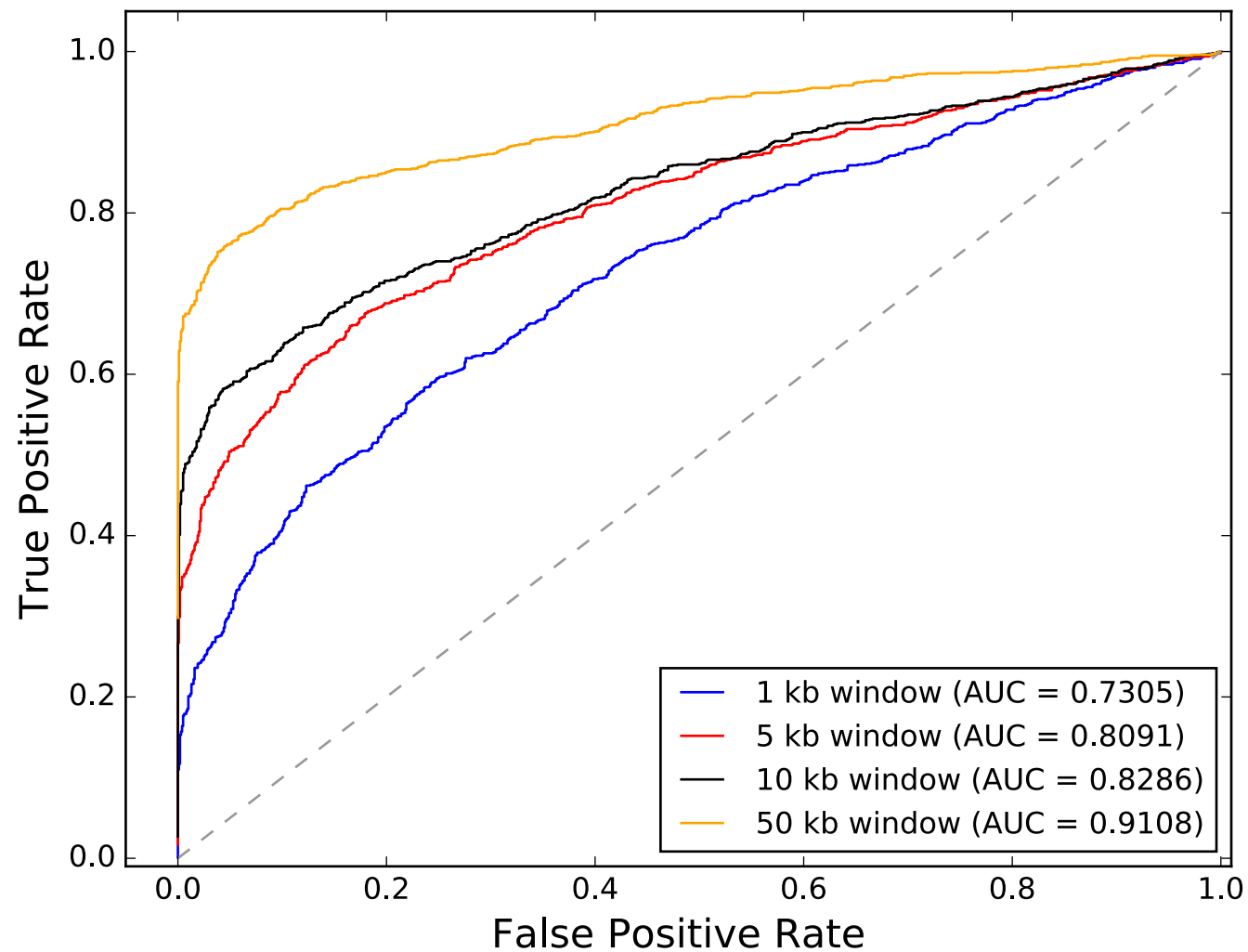

Supplement: S7 Fig — (PDF) [file pgen.1007341.s007.pdf]

Figure S8

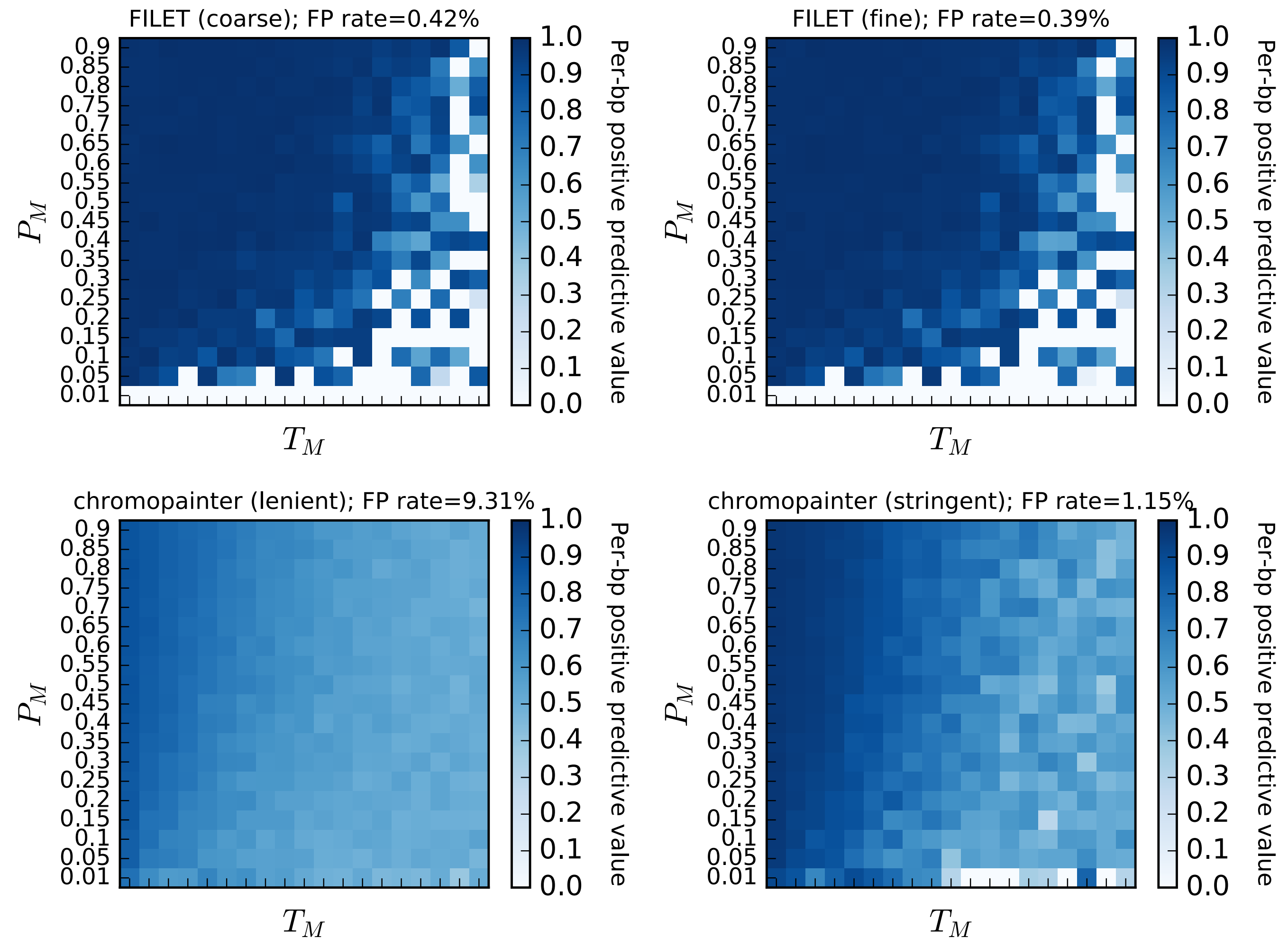

Supplement: S8 Fig — The “coarse” and “fine” versions of FILET, and lenient and stringent versions of ChromoPainter’s predictions, are as defined for Fig 2. In cases where the positive predictive value is undefined (i.e. no base pairs were predicted to be introgressed), it is displayed as zero (i.e. a white cell in the heatmap). (PDF) [file pgen.1007341.s008.pdf]

Figure S9

A

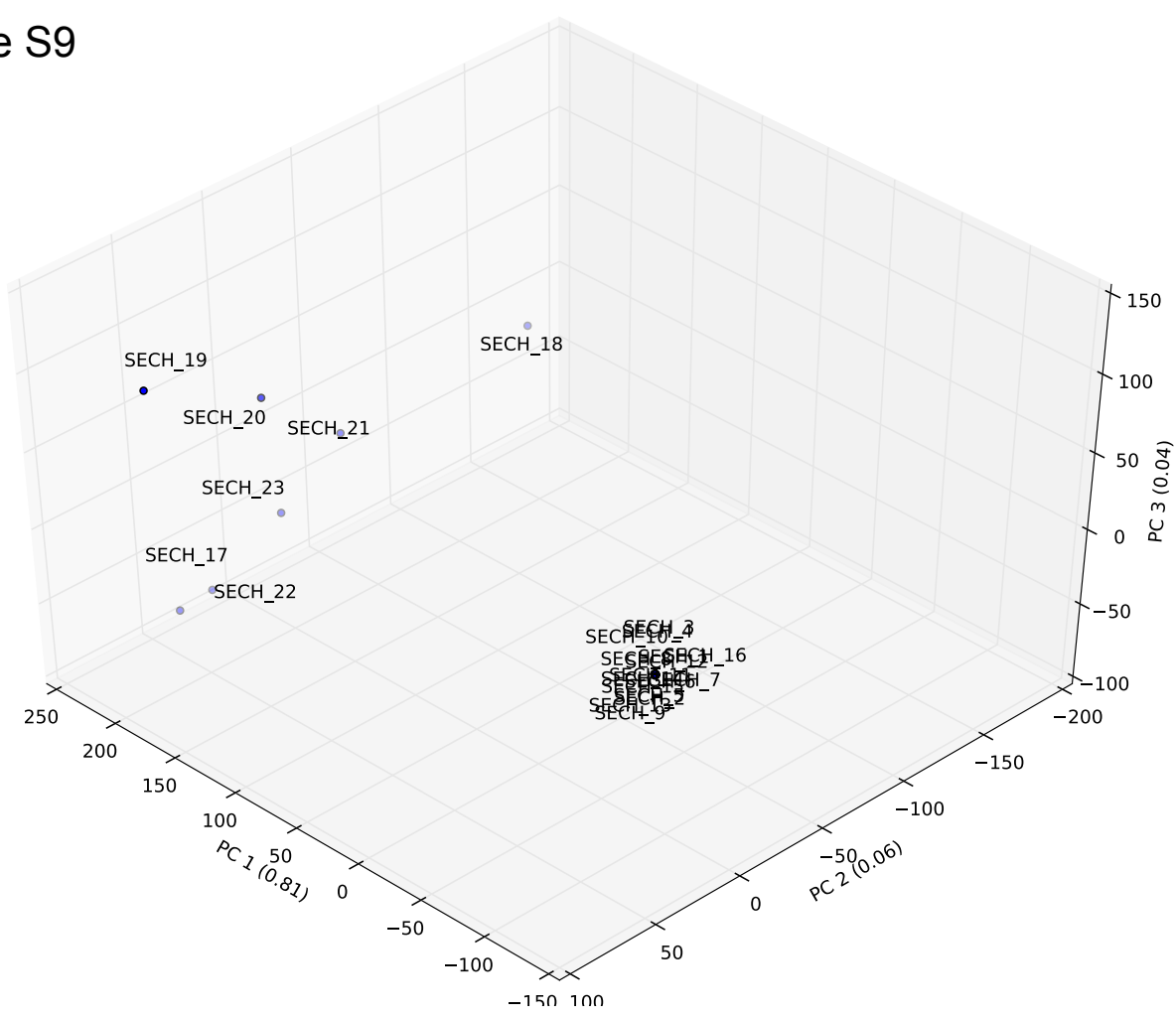

B

K=2

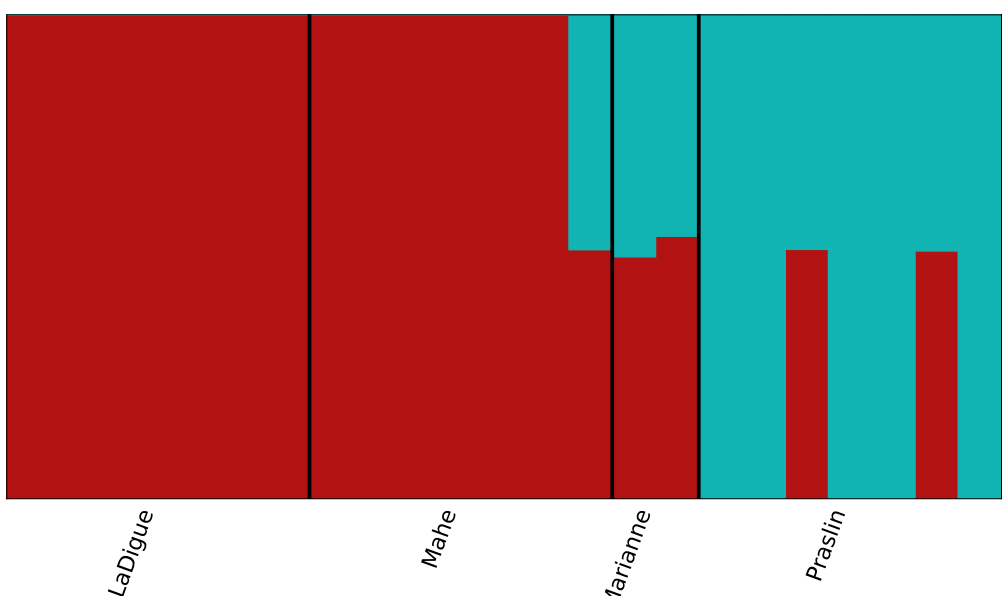

K=3

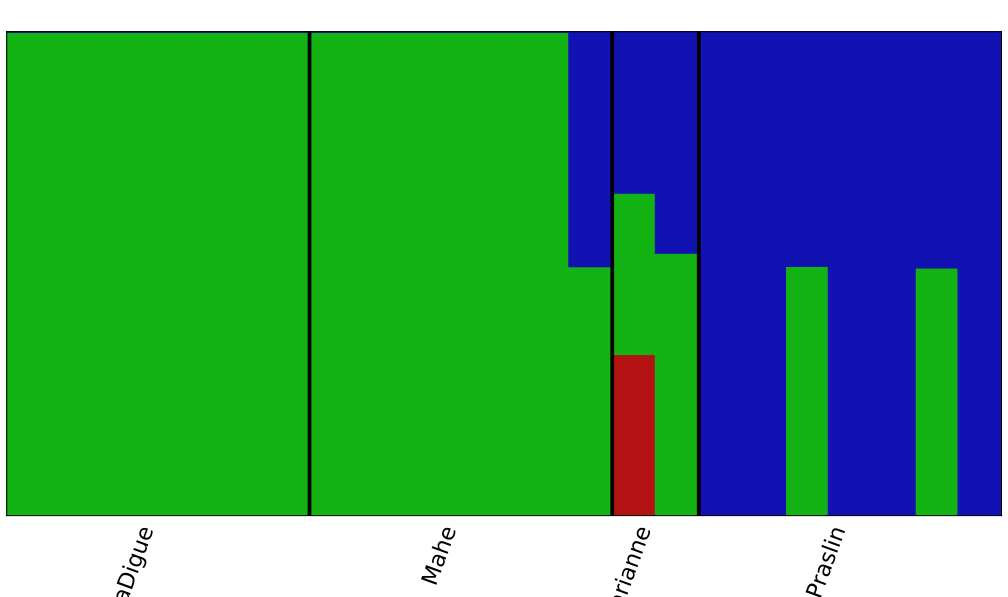

K=4

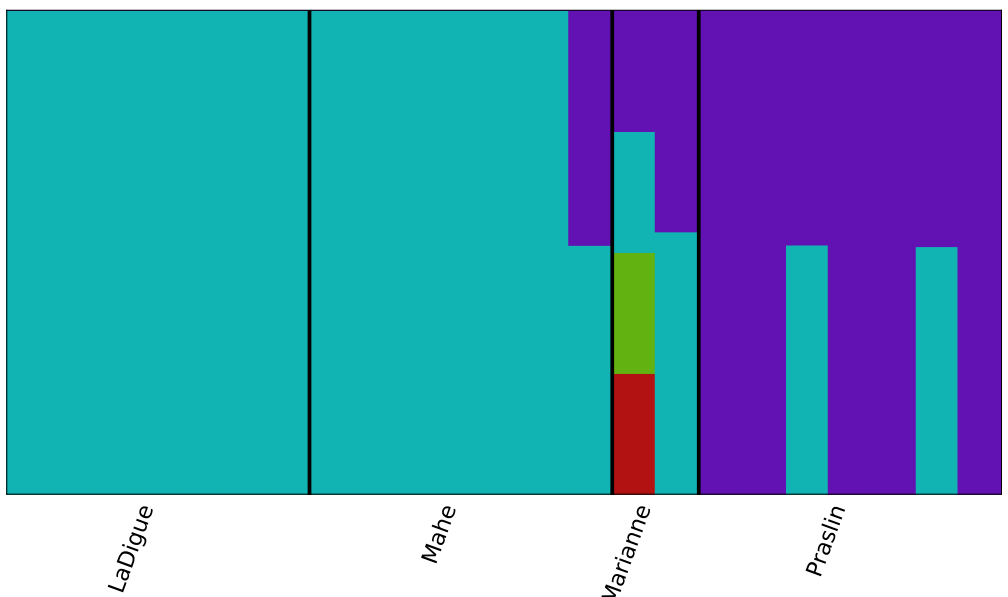

K=5

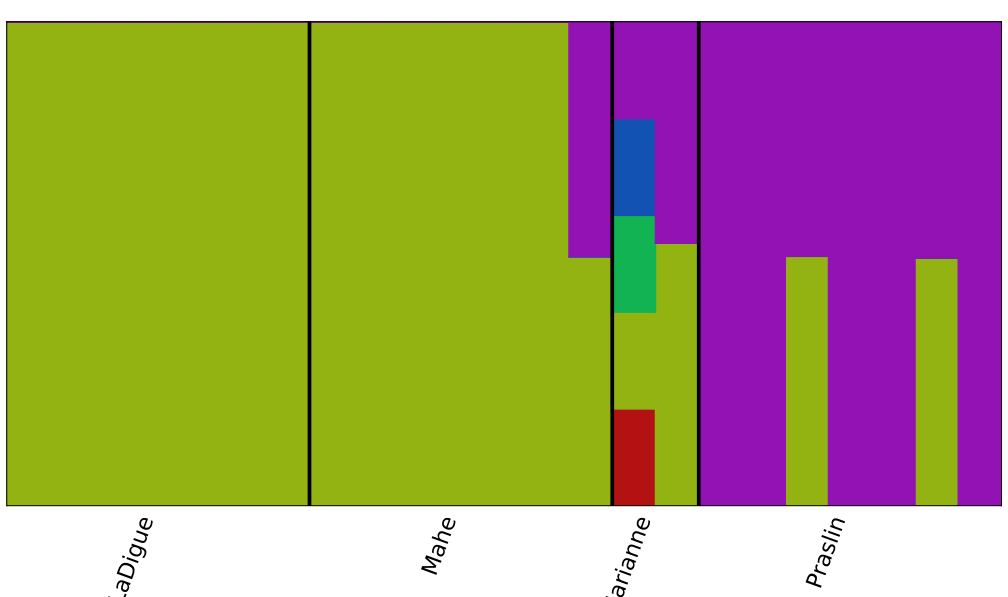

K=6

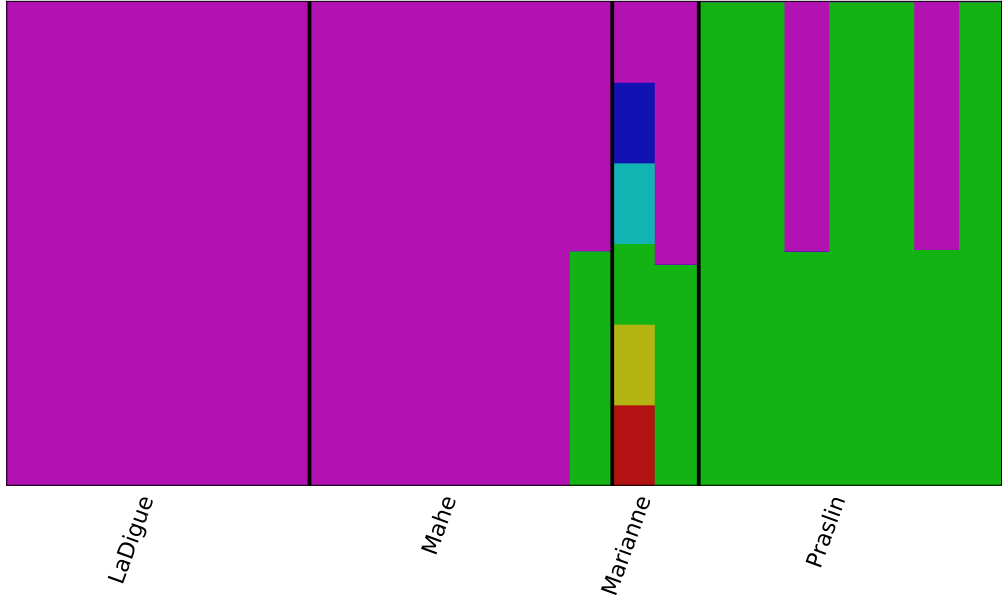

K=7

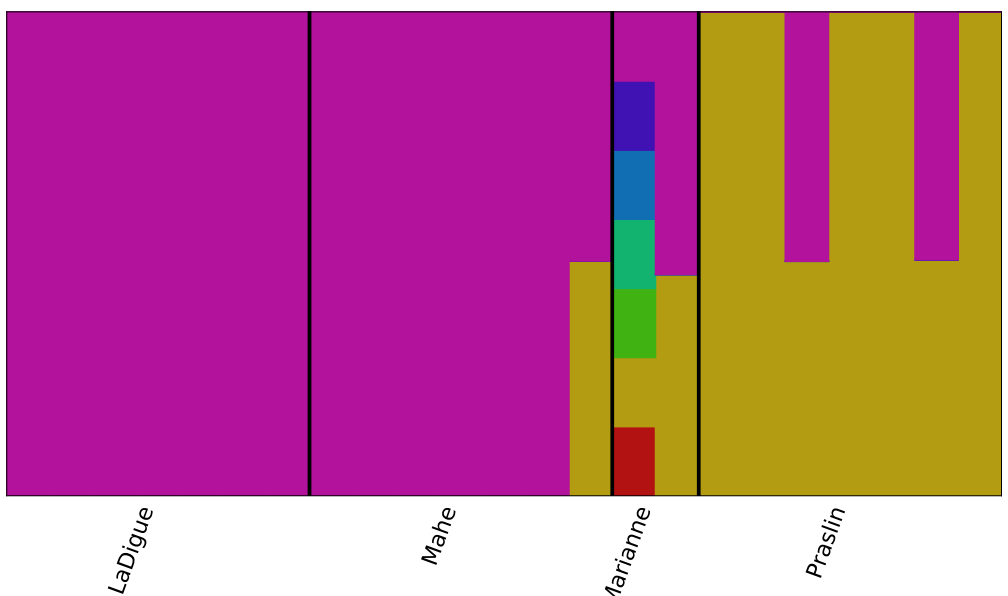

K=8

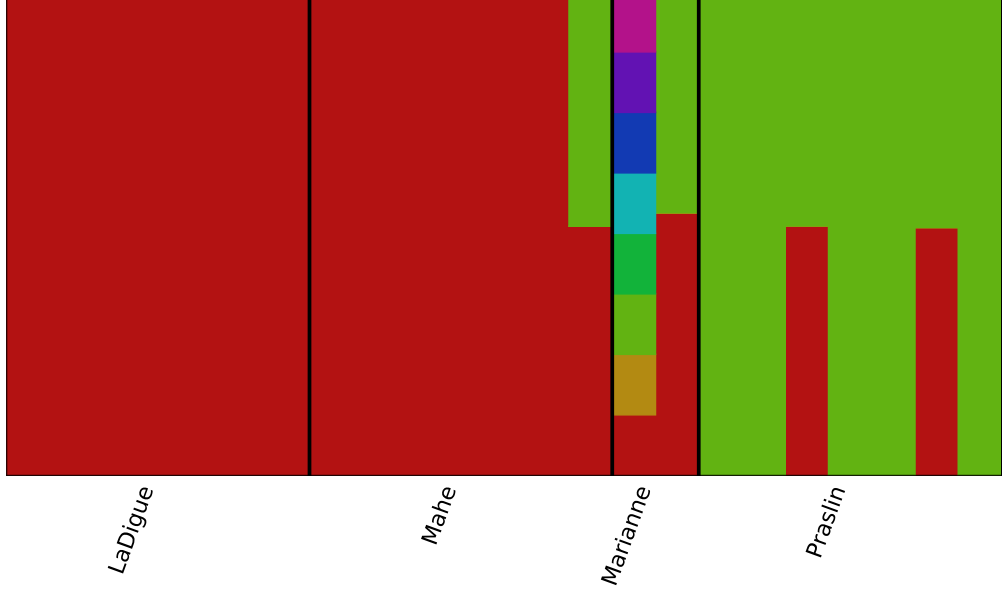

Supplement: S9 Fig — (A) The top three principal components of all D. sechellia diploid genomes. The cluster on the left shows the individuals from Praslin, while the cluster on the right shows all other individuals. Note that the cluster on the right is far less dispersed due to the very small amount of polymorphism among these individuals. The numbers in parentheses on each axis show the fraction of the variance explained by each principal component. (B) Results of running fastStructure on our D. sechellia samples with the number of subpopulations (K) ranging from 2 to 8. (PDF) [file pgen.1007341.s009.pdf]

Figure S10

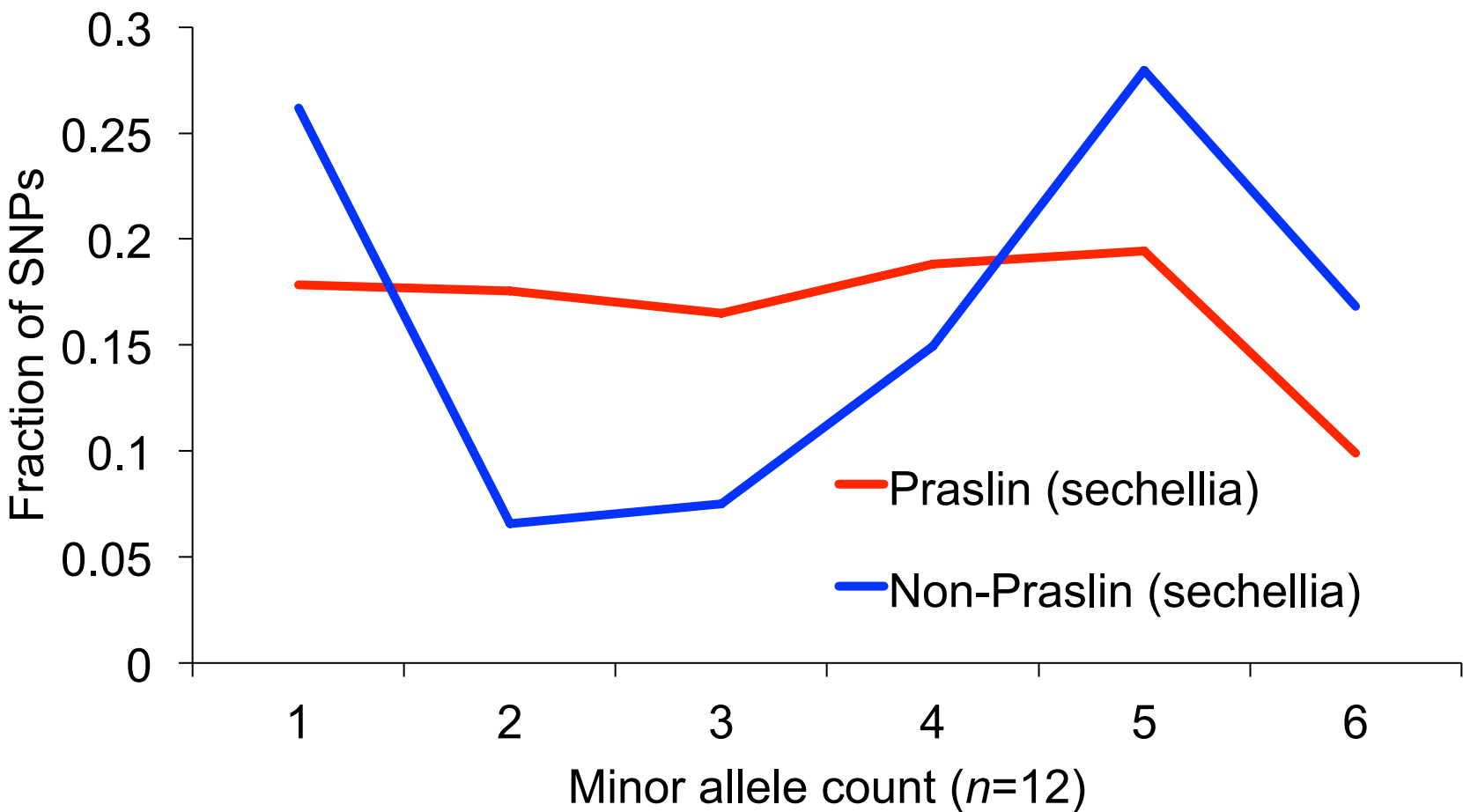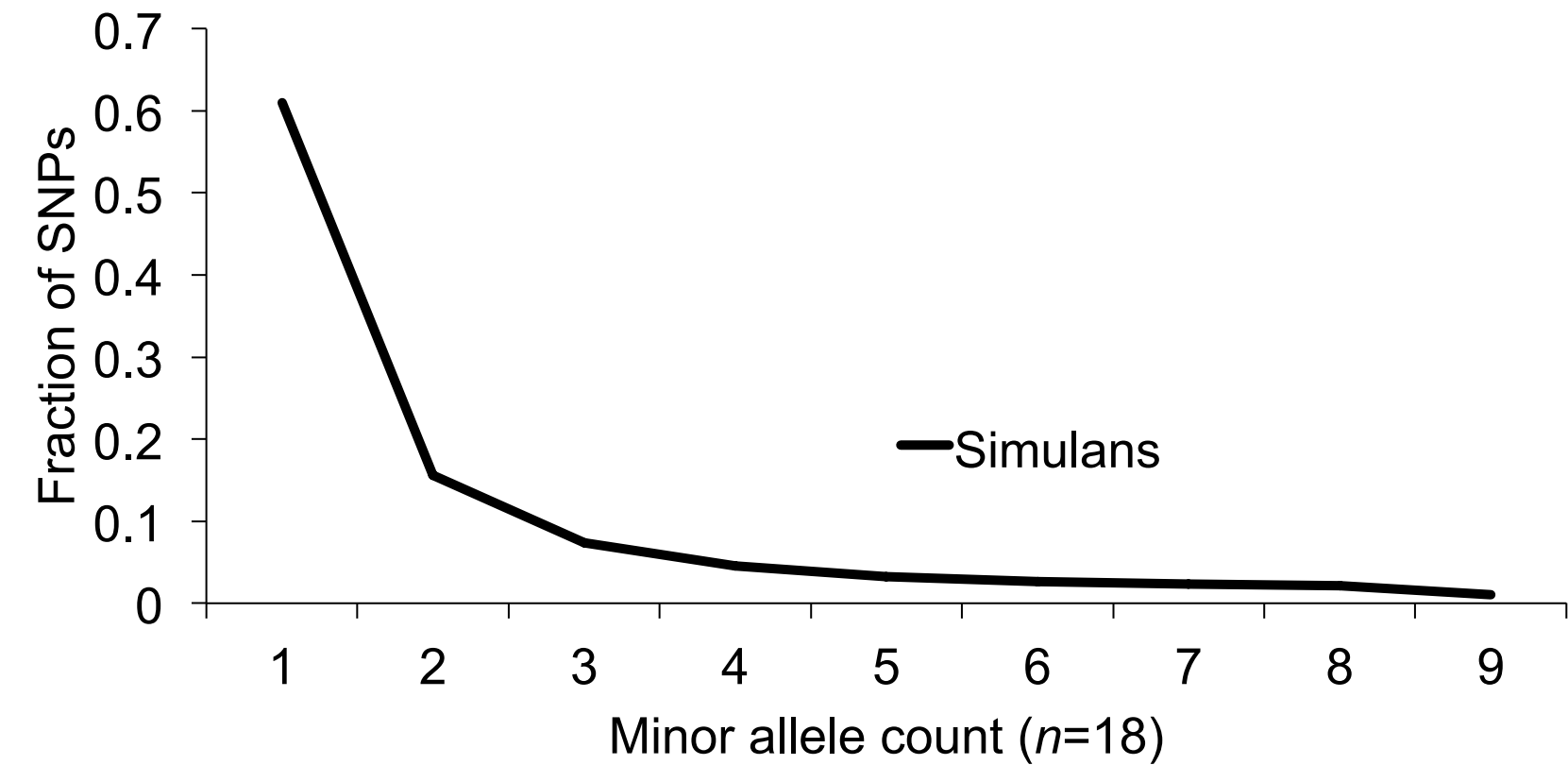

Supplement: S10 Fig — The D. sechellia samples were both downsampled to n = 12 as described in the text, while D. simulans was downsampled to n = 18 (i.e. the same sample sizes used for our demographic inference). These SFS show the fraction of all polymorphisms found in each bin rather than the raw number of polymorphisms, and thus do not contain information about the total number of SNPs. As described in the text, there is >12-fold more polymorphism in the Praslin samples than in the non-Praslin samples. (PDF) [file pgen.1007341.s010.pdf]

Figure S11

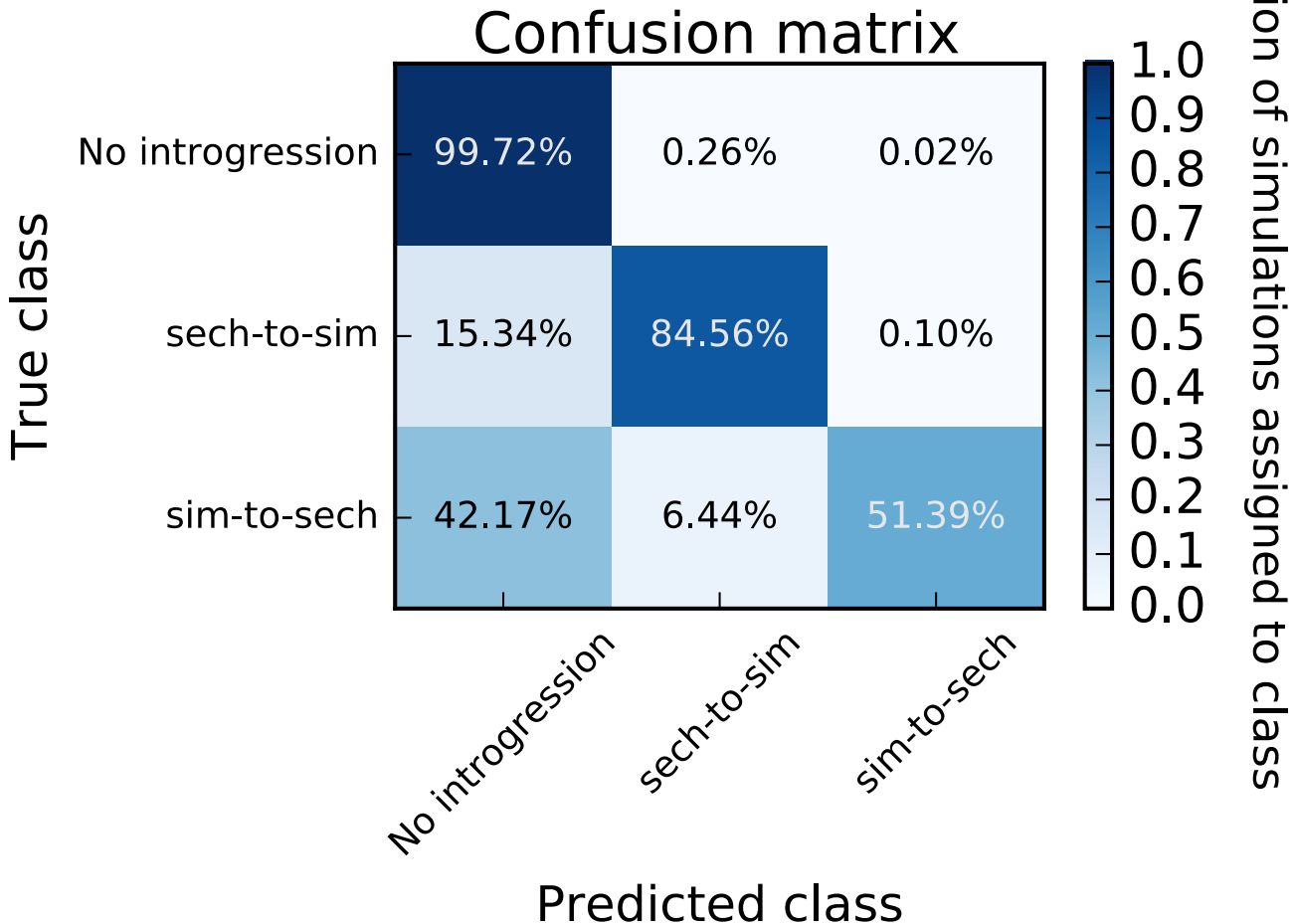

Supplement: S11 Fig — Under this model as assessed on an independent simulated test set. Perfect accuracy would be 100% along the entire diagonal from top-left to bottom-right, and the false positive rate is the sum of top-middle and top-right cells. (PDF) [file pgen.1007341.s011.pdf]
